# Survey data from 38 integrated crop-livestock farming systems in Western France

**DOI:** 10.1016/j.dib.2018.03.066

**Published:** 2018-03-21

**Authors:** Matthieu Carof, Olivier Godinot

**Affiliations:** SAS, AGROCAMPUS OUEST, INRA, 35042 Rennes, France

## Abstract

This paper presents data collected from 38 integrated crop-livestock farming systems in Ille-et-Vilaine, Brittany, France, during face-to-face surveys. Surveys were conducted using a quantitative questionnaire to collect information about farm management practices that affect nitrogen (N) inputs, N outputs, and internal N flows. The data were used to develop new indicators of N efficiency (SyNE, System N Efficiency) and of N balance (SyNB, System N Balance), as described in “SyNE: An improved indicator to assess nitrogen efficiency of farming systems” [1]. Also, the data were used to test an online tool developed to calculate these indicators, as described in “A free online tool to calculate three nitrogen-related indicators for farming systems” [2]. The data are provided with this article.

**Specifications table**

**Table:** 

Subject area	*Agricultural science*
More specific subject area	*Agronomy, Agroecological engineering*
Type of data	*Table*
How data were acquired	*Survey*
Data format	*Raw and analyzed*
Experimental factors	*–*
Experimental features	*–*
Data source location	*Ille-et-Vilaine, Brittany, France*
Data accessibility	*Data are provided with this article*

**Value of the data**

• The data allow researchers to describe nitrogen (N) management (N inputs such as fertilizer and feed purchased; N outputs such as milk and animals sold; internal N flows such as change in soil N stock) in integrated crop-livestock farming systems in Western France.
• The data can be used to calculate indicators of N efficiency and N balance for these integrated crop-livestock farming systems.
• The data can be used to compare crop and livestock management practices from other regions and other farming systems.
• Since all surveyed farmers cropped alfalfa and other legumes in variable proportions, the data can be useful for studying the N self-sufficiency of these systems.

## 1 Data

**Fig. 1:**
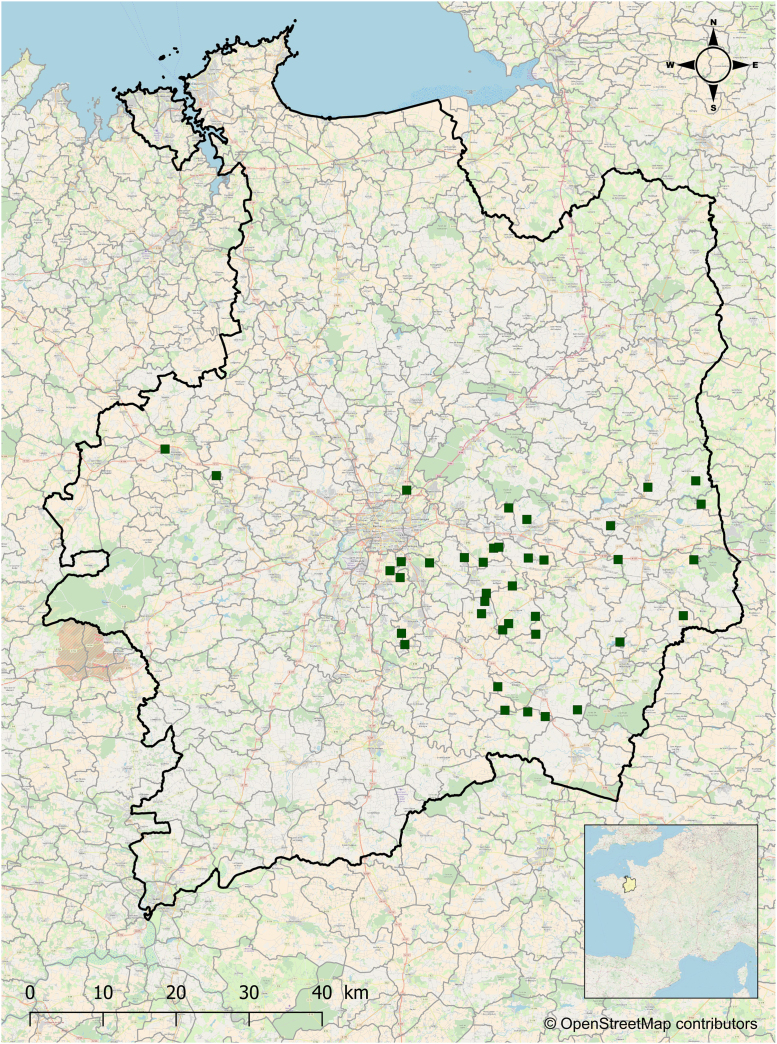
A map of the Ille-et-Vilaine department in Brittany, France, where the data were collected. Brittany is designated as a Nitrate Vulnerable Zone according to the European Union Nitrates Directive [3]. Green squares show locations of the 38 surveyed farming systems. (For interpretation of the references to color in this figure legend, the reader is referred to the web version of this article.)

Thirty-eight integrated crop-livestock^1^ farming systems were surveyed in spring 2012 to develop new indicators of N efficiency [1]. The farming systems were located in the department of Ille-et-Vilaine, eastern Brittany, France, which is designated as a Nitrate Vulnerable Zone according to the European Union (EU) Nitrates Directive [3] (Fig. 1). Contacts were provided by an agricultural cooperative specialized in alfalfa dehydration; therefore, all surveyed farmers cropped alfalfa.

Brittany, a lowland area, is the most important region in France for livestock production (e.g., 21% of national milk production, with an average of 7158 L per cow in 2011 [4]). Crop production is targeted mostly towards livestock feeding and is dominated by winter wheat (17% of regional utilized agricultural area (UAA)), maize (26% of regional UAA), and grasslands (41% of regional UAA) [4].

## 2 Experimental design, materials and methods

The 38 integrated crop-livestock farming systems were surveyed to collect information about their N inputs, N outputs, and internal N flows for the year 2011. A face-to-face survey with each farmer was conducted by a researcher trainee. It was mostly quantitative, with closed questions, and lasted 1 to 2 h. A simplified version of the questionnaire, translated into English, is available as Supplementary material.

Farmers were asked about crop areas and yields, herd composition, sales of animal products and crops, feed and fertilizer purchases, manure management, and other information related to N flows in the farming system. Mean characteristics of the 38 surveyed farming systems are presented in Table 1.

**Table 1:** Descriptive statistics of the 38 surveyed farming systems in Ille-et-Vilaine, Brittany, France.

Variable	*n*	Minimum	Maximum	Mean	Standard deviation
Utilized agricultural area (ha)	38	47.2	367.0	108.3	60.3
Alfalfa area (ha)	38	1.6	23.6	6.5	4.8
Silage maize area (ha)	38	6.0	65.1	25.7	12.6
Winter wheat area (ha)	34	3.0	107.0	27.5	21.3
Dairy cows (number)	38	30	197	70	34
Milk production (kg FPCM^a^)	38	246,001	1,669,098	596,456	286,080
Feed purchase (kg N)	38	244	15 849	4 358	3 471
Inorganic fertilizer purchase (kg N)	35	1 072	35 563	8 684	7 114
Manure purchase (kg N)	28	300	16 470	3 038	3 123

a FPCM, Fat and Protein Corrected Milk.

The available data were refined from the raw data by correcting errors, filling in missing values, and rendering data for each faming system consistent. Values of N inputs, N outputs, and internal N flows were calculated using the free online tool available at https://www.nefficiencycalculator.fr/en/ [2].

## Footnotes

1 In this article, livestock refers only to cattle.
