# Supplementary material for: Survey data from 38 integrated crop-livestock farming systems in Western France
Source: Data Brief. 2018 Mar 21;18:723–6. doi: 10.1016/j.dib.2018.03.066 (PMC5996288; doi:10.1016/j.dib.2018.03.066)
Supplement: Supplementary file 3 — Supplementary material [file mmc3.pdf]

PRESENTATION AND STRUCTURE OF THE FARM

- Would you provide some information about yourself?

Last name	
First name	
Age	
Street address	
Postal code, Town	
Cell phone	
E-mail address	

- Would you describe your farm in a few words (e.g., products produced, specification sheets, quality labels, conventional or organic production, agro-environmental measures)?

- How many people (permanent or temporary) work on your farm (Man Work Units)?

- How large is your farm (ha of Utilized Agricultural Area)?

- Would you describe your farm's soils?
    - Soil type(s)

Topsoil		Bedrock	
Silts		Schist	
Silty-clay loams		Granite	
Medium loams		Sandstone	
Sandy-clay loams		Other	
Other			

-
    - Stone content
    - Organic matter content

- What is your milk quota (in L) and its characteristics in 2011?

Milk quota	
Protein content	
Fat content	

- Do you produce any other animals (please specify)?

ANIMAL PRODUCTION

- What is the composition of your cattle herd in 2011 (breed, number, average liveweight)?

Animal category	Initial stock	Bought	Sold	Self-consumed	Losses	Final stock
Dairy cows						
Dairy heifers, 0-1 years old						
Dairy heifers, 1-2 years old						
Dairy heifers, 2+ years old						
Suckler cows						
Calves sold at 8-15 days						
Animals sold at 0-1 years						
Animals sold at 1-2 years						
Animals sold at 2+ years						
Bulls						
Other						

- How much milk do you sell (in L) in 2011?

- How do you manage your herd?
  - Key dates

Start of grazing	
End of grazing	
Start of night-time grazing	
Start of nights in barns	
Closing of the silo	
Opening of the silo	

- How much animal feed did you buy (in t) in 2011?

Type	Initial stock	Bought	Final stock
**Protein concentrates**			
Soybean meal 48%			
Soybean meal 46%			
Soybean meal 44%			
Canola meal 35%			
Urea			
Dehydrated alfalfa 16% CP			
Dehydrated alfalfa 22% CP			
Compound feed 36% CP			
Compound feed 40% CP			
Compound feed 44% CP			
**Energy concentrates**			
10%			
12%			
14%			
**Production concentrates**			
VL 18%			
VL 22%			
VL 26%			
VL 30%			
**Forages**			
Headed-grass hay			
Ryegrass-white clover hay			
Alfalfa hay			
Cereal straw			
Maize silage			
Wrapped bales			
Other			

CROP PRODUCTION

- What was your farm's crop configuration in 2011?

Crop	Area	Yield	Duration
Permanent pastures			
Pure grass pastures			
Perennial ryegrass			
Annual ryegrass			
Fescue			
Other			
Grass-legume pastures (give % legume)			
Perennial ryegrass-white clover			
Annual ryegrass-red clover			
Hybrid ryegrass-red clover			
Orchard grass-alfalfa			
Other			
Silage maize			
Grain maize			
Winter wheat			
Spring wheat			
Winter barley			
Spring barley			
Triticale			
Oats			
Forage beets			
Alfalfa			
Canola			
Peas			
Field bean			
Lupine			
Cereal mixture			
Other			

- What are your main crop rotations?
- Do you plant any intercrops or catch crops (please specify)?
- How do you manage crop residues (straw)?

- Did you sell some of your crops in 2011? If so, how much (in t)?

Category and crop	Initial stock	Bought	Sold	Final stock
**Cereals and oilseed/protein crops**				
Grain maize				
Winter wheat				
Spring wheat				
Winter barley				
Spring barley				
Triticale				
Oats				
Canola				
Peas				
Field beans				
Lupine				
**Forages**				
Silage maize				
Forage beets				
Headed-grass hay				
Perennial ryegrass-white clover hay				
Pre-heading legume hay				
Alfalfa hay				
Permanent pasture grass				
Fine-cut grass silage				
Haylage				
Forage canola				
Ammonia-treated straw				
Untreated straw				
Other				

FERTILISATION

- Did you import and export organic fertilizer in 2011 (in t)?

Type	Imported	Exported
Manure		
Slurry		
Other		
	Yes (amount)	No
Straw/manure exchanges		

- Did you buy inorganic fertilizer in 2011 (in t)?

Inorganic fertilizer	Initial stock	Bought	Final stock
**Nitrogen fertilizers**			
Ammonium nitrate			
Anhydrous ammonia			
Ammonium sulfate			
Urea - granulated urea			
Diammonium phosphate (18-46 maize starter)			
Calcium cyanamide			
Calcium nitrate			
Potassium nitrate			
**Phosphate fertilizers**			
Triple superphosphate (45%)			
Superphosphate (18%)			
Thomas slag			
**Potassium fertilizers**			
Potassium chloride			
Potassium sulfate			
**Others**			

- Would you provide some characteristics of your barns?

	Dairy cows	Heifers 0-1 years	Heifers 1-2 years	Heifers 2+ years
Type*				
kg straw/animal/day				
***1**	Barn with bays, compact manure			
**2**	Straw yard, manure scraping, soft manure			
**3**	Head-to-head stalls, compact manure scraping			
**4**	Back-to-back stalls, scraped feeding alley, manure			
**5**	Head-to-head stalls, soft manure scraping			
**6**	Barn with bays, soft manure			
**7**	Straw yard, all slurry scraping			
**8**	Straw yard, manure scraping, compact manure			
**9**	Back-to-back stalls, scraped feeding alley, slurry (+ cow “niches”)			
**10**	Stalls with slatted floors (+ all other systems with 100% slurry)			
**11**	Head-to-head stalls, slurry scraping			
**12**	Accumulated litter			

- Area available per cow
